# Beneficial Impact of Nutritional Therapy on Idiopathic Pulmonary Fibrosis

**DOI:** 10.7759/cureus.78594

**Published:** 2025-02-05

**Authors:** Hideaki Yamakawa, Hiroki Ohta, Shintaro Sato, Hidekazu Matsushima

**Affiliations:** 1 Respiratory Medicine, Saitama Red Cross Hospital, Saitama, JPN

**Keywords:** drug therapy, idiopathic pulmonary fibrosis, interstitial lung disease, nintedanib, nutrition therapy

## Abstract

Although several studies have reported that poor nutritional status is associated with a worse prognosis in patients with interstitial lung disease (ILD), the beneficial impact of nutritional therapy has not yet been established. We report a case of idiopathic pulmonary fibrosis (IPF) in which nutritional therapy played an important role alongside drug therapy. A 71-year-old Japanese male was diagnosed with IPF and started on nintedanib. However, he experienced appetite loss, leading to significant weight loss and disease progression. Consequently, nintedanib was discontinued, and a dietitian introduced a high-fat, high-protein nutritional therapy. His condition improved, allowing nintedanib to be restarted after a period of cessation. Following multiple nutritional education sessions, his condition stabilized without further appetite loss. These findings suggest that when determining treatment strategies for patients with ILD, clinicians should incorporate appropriate nutritional management during long-term treatment with effective anti-ILD agents to optimize patient outcomes.

## Introduction

In idiopathic pulmonary fibrosis (IPF), multiple factors can adversely affect nutritional well-being, such as heightened strain on respiratory muscles, the release of inflammatory chemical messengers, coexistent low blood oxygen levels, and a lack of physical exercise [1]. Although antifibrotic agents such as nintedanib and pirfenidone have shown a favorable impact by significantly delaying the decline in pulmonary function in IPF, research has suggested that Asian patients experience a lower rate of continued use of antifibrotic medications [2-4]. As background information, low body weight and weight loss were significant predictors of discontinuation of antifibrotic agents [5,6]. However, because prolonged use of antifibrotic agents can result in a more favorable outcome, appropriate management of patient physique is necessary during long-term use; therefore, nutritional therapy, in addition to an antifibrotic agent, is crucial [6]. We herein report the case of a patient with IPF who received effective nutritional therapy during nintedanib treatment.

## Case presentation

**Figure 1:**
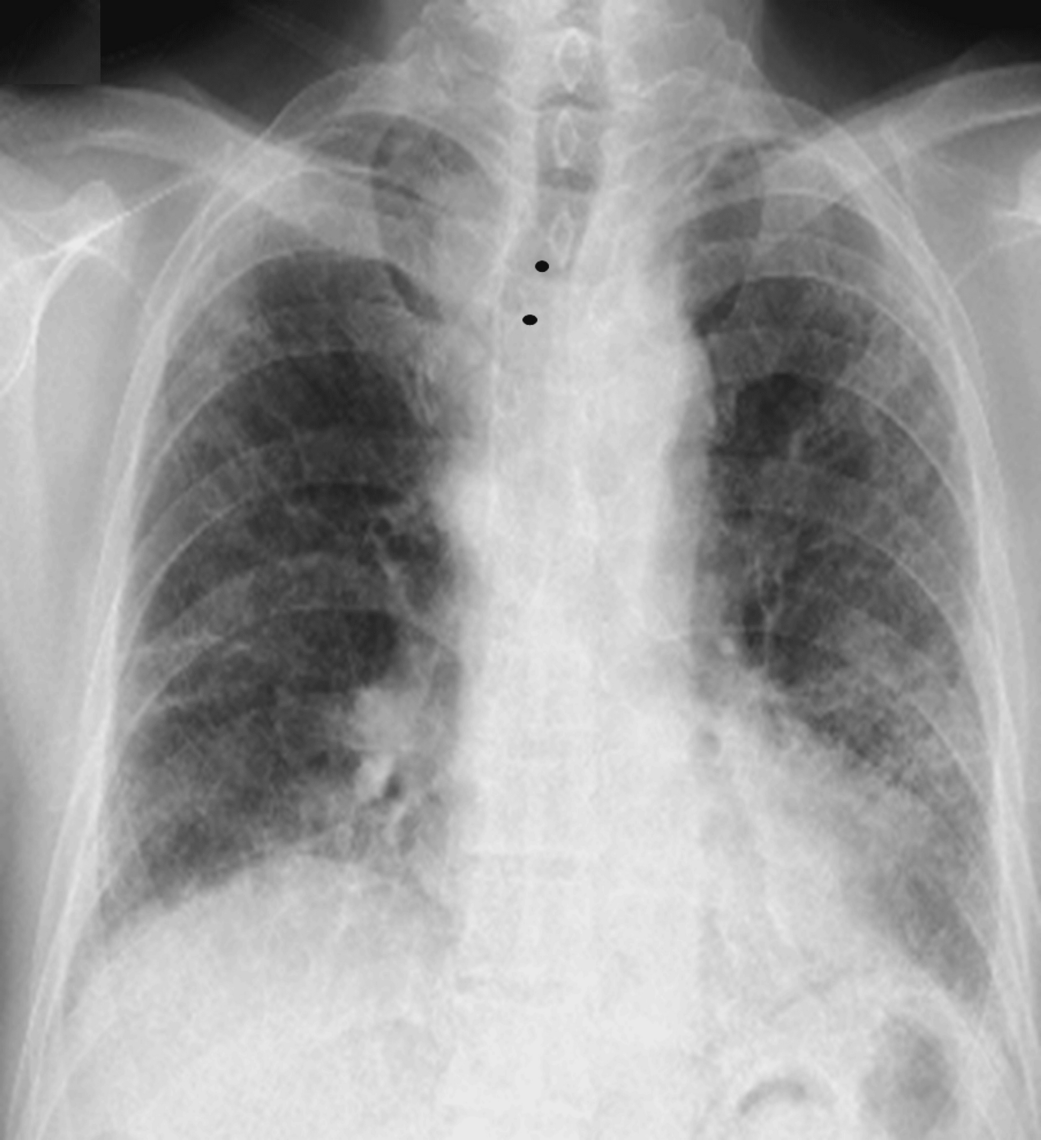
Chest X-ray at initial visit A chest X-ray showed volume loss, tracheal deviation (black circles), and bilateral reticulation predominantly in the lower lung.

The patient, a 71-year-old Japanese male ex-smoker, was admitted to our hospital with dyspnea on exertion in March 2022. Fine crackles were audible at the bases of both lungs during inspiration. A chest X-ray showed volume loss and bilateral reticulation, predominantly in the lower lungs (Figure 1).

**Figure 2:**
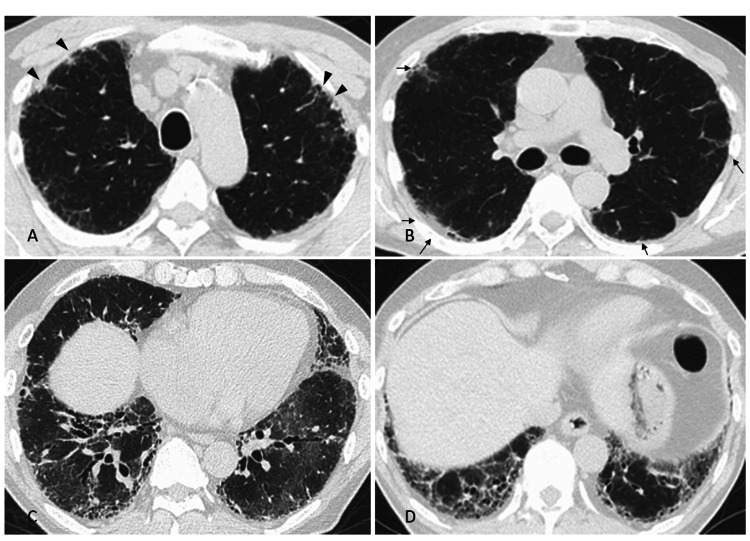
Chest computed tomography images (A, B) In the upper-lung zone, bilateral reticulation (arrows) and irregular thickening along the pleura (arrowheads) were shown. (C, D) Reticular shadows and ground-glass opacities were present predominantly at the base of both the lungs.

A chest computed tomography (CT) scan revealed reticulation and ground-glass opacifications at the bases of both lungs (Figure 2).

A physical examination revealed no suspicion of connective tissue disease (CTD), which is typically accompanied by symptoms such as Raynaud's phenomenon, skin thickening on the fingers of both hands, nail fold capillary bleeding, xerophthalmia, or xerostomia. Assessment of autoantibodies for each CTD in the serum also showed no positive findings. As shown in Table 1, blood tests revealed that the patient's levels of KL-6 and SP-D were significantly high at 3,360 U/mL and 776 ng/mL, respectively.

**Table 1:** Laboratory findings at initial visit WBC: white blood cell, LDH: lactate dehydrogenase, AST: aspartate aminotransferase, ALT: alanine aminotransferase, BUN: blood urea nitrogen, Cr: creatinine, CK: creatine kinase, CRP: C-reactive protein, KL-6: Krebs von den Lungen-6, SP-D: surfactant protein-D, FVC: forced vital capacity, %FVC: percentage of predicted forced vital capacity, FEV1: forced expiratory volume in one second, FEV1/FVC: ratio of forced expiratory volume in one second to forced vital capacity, DLCO: diffusing capacity of the lungs for carbon monoxide, %DLCO: percentage of predicted diffusing capacity of the lungs for carbon monoxide.

Labs	Observed value	Reference range
White blood cell	9860/μL	3300-8600/μL
LDH	422 U/L	124-222 U/L
AST	47 U/L	13-30 U/L
ALT	22 U/L	10-42 U/L
BUN	22.6 mg/dL	8-20 mg/dL
Cr	1.25 mg/dL	0.65-1.07 mg/dL
CK	96 U/L	59-248 U/L
CRP	0.6 mg/dL	<0.14 mg/dL
KL-6	3360 U/mL	<500 U/mL
SP-D	776 ng/mL	<110 ng/mL
Rheumatoid factor	<3 IU/mL	<3 IU/mL
Anti-nuclear antibodies	×80 (Homogeneous ＆ Speckled)	
Anti-SS-A antibody	<1.0 U/mL	<1.0 U/mL
Anti-aminoacyl-tRNA synthetase antibody	<5 units	<25 units
Respiratory function tests		
FVC	1.98 L	3.36 L (predicted value)
%FVC	58.9%	-
FEV_1_	1.83 L	2.54 L (predicted value)
FEV_1_/FVC ratio	92.4%	-
DL_CO_	6.83 mL/min/Torr	16.35 mL/min/Torr (predicted value)
%DL_CO_	41.8%	-

Results from respiratory function tests indicated restrictive lung function issues, as evidenced by the following: a forced vital capacity (FVC) of 1.98 L, representing 58.9% of predicted values; a forced expiratory volume in one second (FEV₁) of 1.83 L; an FEV₁/FVC ratio of 92.4%; a diffusing capacity of carbon monoxide (DLCO) of 6.83 mL/min/Torr, corresponding to 41.8% of predicted values; and a DLCO/alveolar volume ratio of 78.5% of predicted values. He was diagnosed with IPF based on these findings and was then followed up and treated with nintedanib as the antifibrotic agent.

**Figure 3:**
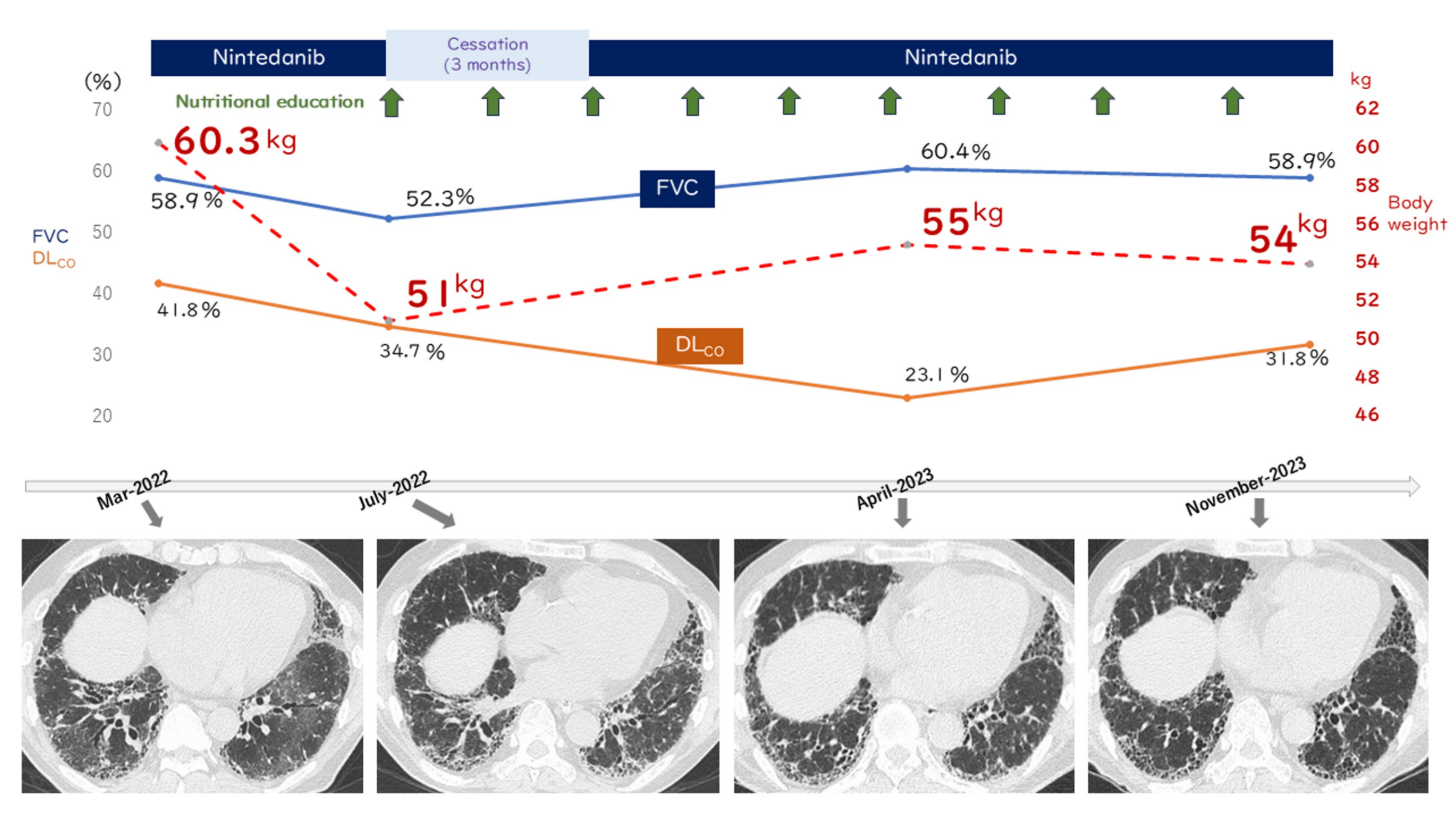
Clinical and radiological course Patient's clinical course. The patient's initial weight was 60.3 kg, and respiratory function tests revealed restrictive ventilatory impairment, with a forced vital capacity (FVC) measured at 1.98 L, or 58.9% of predicted values; a forced expiratory volume in one second (FEV₁) recorded at 1.83 L; an FEV₁/FVC ratio of 92.4%; a diffusing capacity of carbon monoxide (DLCO) of 6.83 mL/min/Torr, or 41.8% of predicted values; and a DLCO/alveolar volume ratio of 78.5% of predicted values. He was administered nintedanib following a diagnosis of idiopathic pulmonary fibrosis. Subsequently, he complained of appetite loss. Symptomatic treatment did not improve his condition, and after four months, his body weight in July 2023 had dropped by 9.3 kg to 51 kg. The extent of the disease had progressed on chest CT, and his pulmonary function parameters had also worsened (FVC to 52.3% and %DLCO to 34.7%). Nintedanib was discontinued for three months, and the dietitian nutritionist initiated an intervention with nutritional therapy. His condition gradually improved, and nintedanib was readministered after a three-month cessation. By April 2023, his body weight had increased by 4 kg to 55 kg, FVC had increased to 60.4% (although %DLCO decreased to 23.1%), and the extent of lung disease had stabilized. By November 2023, after nine sessions of nutritional education, his general condition, pulmonary function, and chest CT findings had remained stable.

Subsequently, he complained of appetite loss. Symptomatic treatment did not improve his condition. After four months, his body weight in July 2023 had dropped by 9.3 kg to 51 kg from an initial weight of 60.3 kg (Figure 3).

The chest CT revealed disease progression, and his pulmonary function had also worsened (FVC to 52.3% and %DLCO to 34.7%). Therefore, nintedanib was discontinued, considering the possibility that appetite loss was caused by nintedanib itself and not solely by interstitial lung disease (ILD). In addition, a dietitian nutritionist initiated an intervention with nutritional therapy. Specifically, the dietitian nutritionist recommended a high-fat, high-protein diet with moderate carbohydrates, based on nutritional therapy for patients with chronic obstructive pulmonary disease (COPD) [7]. Detailed recommendations included using fat oil to improve efficient calorie uptake (e.g., fried rice, tempura, rice bowls, fried chicken, mayonnaise); consuming branched-chain amino acids to maintain or build muscle (e.g., chicken breast, tuna, bonito, mackerel, Pacific saury, soy, cheese, milk); incorporating omega-3 fatty acids for their anti-inflammatory effects (e.g., perilla oil, flaxseed oil); taking oral nutritional supplements; and eating between meals to improve nutritional intake. Initial nutritional education took about 40 minutes, followed by repeated sessions lasting 20 minutes at 1- to 2-month intervals. As his condition gradually improved, nintedanib was reintroduced after a three-month cessation. With continued nintedanib treatment supported by nutritional therapy, he gained 4 kg (reaching 55 kg), his FVC increased to 60.4% (although his %DLCO decreased to 23.1%), and the extent of his lung disease had stabilized by April 2023. By November 2023, after nine nutritional education sessions and without experiencing appetite loss upon restarting nintedanib, his general condition, pulmonary function, and chest CT findings had remained stable. Although nutritional guidance was continued, his ILD became progressive again around February 2024, with gradually worsening dyspnea, and he died in July 2024 from respiratory failure associated with the chronic progression of ILD.

## Discussion

In this report, we described a patient with IPF who experienced weight loss after nintedanib administration. Nutritional therapy provided by the dietitian nutritionist was considered highly effective for this patient and, moreover, facilitated the long-term use of nintedanib. This important report highlights a valuable case based on concrete results of nutritional education.

In patients with IPF, treatment with nintedanib has not been associated with serious adverse events but has been linked to increased gastrointestinal events such as diarrhea, appetite loss, nausea, and weight loss [2,4]. Taking loperamide in conjunction with appropriate nutrition has been recommended for managing diarrhea without compromising the effectiveness of antifibrotic treatment [1,8]. According to Jouneau et al., proactively managing adverse events associated with nintedanib is crucial for patients undergoing nintedanib treatment [9]. When considered collectively, the adverse effects of nintedanib itself, rather than those solely caused by ILD, may contribute to weight loss and potentially lead to a worse prognosis than if nintedanib were not prescribed at all [6].

Because weight loss in individuals with ILD is often linked to more severe disease progression and a worse prognosis [9,10], dietitian nutritionists at our hospital emphasize the importance of maintaining or gaining weight in ILD patients who are normal or underweight, based on previous reports of nutritional support in advanced COPD patients [7]. They typically target a body weight increase of 3-5 kg in underweight patients, as in our case, and then propose the required amount of nutritional intake in a diet comprising high fat (35-50 energy%) and high protein (15-20 energy%) with moderate carbohydrates (30-50 energy%) [7]. Through repeated patient education, patients not only gain body weight but also experience an increase in FVC, and the extent of their lung disease stabilizes. This instructive disease course was suggested to our patient to maintain a physique highly likely to improve prognosis in addition to drug therapy.

## Conclusions

We report the case of a patient with IPF who demonstrated a visible positive effect of nutritional therapy during nintedanib treatment. Low body weight and weight loss can be reasons for discontinuing antifibrotic agents, potentially leading to a poor prognosis. Therefore, chest physicians should be aware that appropriate management of the patient’s physique is necessary during long-term use of antifibrotic agents and that nutritional therapy is equally important as the antifibrotic agent used to treat the patient. In the future, it would be ideal to collect more cases and determine the required total calorie intake for patients with varying severity levels.
